# Green Synthesis of CeO_2_ Nanoparticles from the *Abelmoschus esculentus* Extract: Evaluation of Antioxidant, Anticancer, Antibacterial, and Wound-Healing Activities

**DOI:** 10.3390/molecules26154659

**Published:** 2021-07-31

**Authors:** Hafiz Ejaz Ahmed, Yasir Iqbal, Muhammad Hammad Aziz, Muhammad Atif, Zahida Batool, Atif Hanif, Nafeesah Yaqub, W. A. Farooq, Shafiq Ahmad, Amanullah Fatehmulla, Hijaz Ahmad

**Affiliations:** 1 Institute of Physics: The Islamia University of Bahawalpur, Bahawalpur 63100, Pakistan; mejaz.ahmed@iub.edu.pk (H.E.A.); zahida.batool@iub.edu.pk (Z.B.); 2 Department of Physics, COMSATS University Islamabad, Lahore 54000, Pakistan; yasiriqbal485@gmail.com; 3 Department of Physics and Astronomy, College of Science, King Saud University, P.O. Box 2455, Riyadh 11451, Saudi Arabia; muhatif@ksu.edu.sa (M.A.); 437203488@ksu.edu.sa (N.Y.); wafarooq@hotmail.com (W.A.F.); aman@ksu.edu.sa (A.F.); 4 Botany and Microbiology Department, College of Science, King Saud University, Riyadh 11451, Saudi Arabia; ahchaudhry@ksu.edu.sa; 5 Industrial Engineering Department, College of Engineering, King Saud University, P.O. Box 800, Riyadh 11421, Saudi Arabia; ashafiq@ksu.edu.sa; 6 Section of Mathematics, International Telematic University Uninettuno, Corso Vittorio Emanuele II, 39, 00186 Roma, Italy; hijaz555@gmail.com

**Keywords:** green synthesis, nanobiotechnology, antibacterial activity, wound healing, antioxidant

## Abstract

Metal oxide nanoparticles synthesized by the biological method represent the most recent research in nanotechnology. This study reports the rapid and ecofriendly approach for the synthesis of CeO_2_ nanoparticles mediated using the *Abelmoschus esculentus* extract. The medicinal plant extract acts as both a reducing and stabilizing agent. The characterization of CeO_2_ NPs was performed by scanning electron microscopy (SEM), X-ray diffraction (XRD), ultraviolet-visible spectroscopy (UV-Vis), and Fourier transform infrared spectroscopy (FTIR). The in vitro cytotoxicity of green synthesized CeO_2_ was assessed against cervical cancerous cells (HeLa). The exposure of CeO_2_ to HeLa cells at 10–125 µg/mL caused a loss in cellular viability against cervical cancerous cells in a dose-dependent manner. The antibacterial activity of the CeO_2_ was assessed against *S. aureus* and *K. pneumonia*. A significant improvement in wound-healing progression was observed when cerium oxide nanoparticles were incorporated into the chitosan hydrogel membrane as a wound dressing.

## 1. Introduction

Nanobiotechnology is an advanced and emerging field of science and technology consist of metals and metal oxides at the nano range. In addition, nanobiotechnology has many applications, especially in the biomedical field [1,2,3]. CeO_2_ nanoparticles (NPs) have attained significant consideration in nanomedicine because of their promising applications in drug delivery, catalysis, biosensing, and medicine. CeO_2_ nanoparticles are relatively stable, have exceptional biocompatibility with little or no toxicity, low cost, and are environmentally friendly [4]. Cerium has two oxidation states, e.g., tetravalent (Ce^4+^) and trivalent (Ce^3+^). Therefore, cerium oxide exists as two different oxides ((i.e., Ce_2_O_3_ (Ce^3+^) and CeO_2_ (Ce^4+^)) depending on the nature of the materials [4,5]. CeO_2_ NPs have a cubic fluorite structure, and Ce^3+^ and Ce^4+^ co-occur on their surface. 

The major benefit of cerium dioxide is to produce an oxygen vacancy in the lattice [6,7]. Therefore, the redox properties of CeO_2_ NPs have been improved and are helpful for various diseases associated with oxidative stress problems. Moreover, cerium oxide nanoparticles can produce several reactive oxygen species (ROS) that are crucial for in vitro activities [8,9]. Wound healing is the main part of the inflammatory progression that is composed of several physical procedures that depend on the corresponding functions of vascular endothelial growth factor (VEGF). The integrity of the skin can be restored by the healing of wounds caused by incision or accidental injuries [10,11]. Numerous factors, such as diabetes, can delay the process of wound healing, which slows down the blood flow and angiogenesis process at the wound site [12]. Currently, a polymer-based wound dressing impregnated with metal oxide nanoparticles has been used to treat various types of wounds. ROS produced by the metal oxide nanoparticles stimulate the angiogenesis process [12,13,14].

*Abelmoschus esculentus* has been used for quite a while as a consumable vegetable in various countries. It contains proteins, enzymes, vitamins, and carbohydrates. *Abelmoschus esculentus* exhibits anticancer, antidiabetic, and antifungal activities, owing to its high free radical antioxidant activity [15,16]. *Abelmoschus esculentus* is an excellent source of flavonoids and polysaccharides and is associated with immune system modulation. Numerous studies have indicated that the biomolecules in the *Abelmoschus esculentus* (okra) extract (e.g., terpenoids, alkaloids, tannins, and flavonoids) are essential in the abatement of metals ions and eventual stabilization of nanoparticles [17].

Various studies have been reported chemical and physical approaches for preparing metal and metal oxide nanoparticles in a very short period of time. In chemicals synthesis methods, a toxic material can be adsorbed at the surface of nanoparticles that may cause adverse effects in a biological environment [18,19]. Currently, plant-mediated preparation of metallic nanoparticles is gaining importance because the preparation is simple and eco-friendly, and the extract of these plants consists of different biomolecules such as vitamins, proteins, surfactants, and carbohydrates, which help to stabilize the nanoparticles [20,21]. Therefore, in this study, CeO_2_ nanoparticles were prepared by green synthesis using the *Abelmoschus esculentus* extract. The components existing in the *Abelmoschus esculentus* extract are used as effective capping and reducing agents. Moreover, this study evaluated the cytotoxicity of CeO_2_ NPs on cervical cancerous cells and showed extensive antioxidant activity. In addition, this study aimed to investigate the healing response on a cutaneous wound using green synthesized CeO_2_ nanoparticles incorporated with chitosan hydrogel membrane. 

## 2. Materials and Methods

### 2.1. Materials

Chitosan (degree of deacetylation: 85%), cerium nitrate hexahydrate (Ce(NO_3_)_3_·6H_2_O), ethanol (C_2_H_5_OH), glycerol, 2,2-diphenyl-1-picrylhydrazyl (DPPH), acetic acid (CH_3_COOH), and sodium hydroxide (NaOH)] were purchased from Sigma Aldrich (Schnelldorf, Germany). Deionized and distilled water were used throughout the experiment. The bacterial strains of S. aureus and K. pneumonia were obtained from The Islamia University of the Bahawalpur, Bahawalpur, Pakistan.

### 2.2. Green Synthesis of CeO_2_ Nanoparticles

Fresh fruits of *Abelmoschus esculentus* (okra) were obtained, thoroughly washed with running tap water, and then cut into small parts. Then, 20 g of okra fruit pieces were immersed in 150 mL of distilled water overnight at room temperature. Then, the okra fruit solution was extracted using a Whatman No.1 filter paper. Afterward, a 0.5 M solution of cerium nitrate hexahydrate [Ce(NO_3_)_3_·6H_2_O] was prepared in deionized water, and a further 10 mL of the okra extract was mixed up into the precursor solution. At that time, a prepared 2 M sodium hydroxide (NaOH) solution was added to the mixture dropwise. Then, the solution was centrifuged and washed with distilled water three times and then ethanol. Furthermore, the obtained precipitates were dried at 120 ℃ for 6 h, calcinated for 4 h at 600 ℃ to evaporate water, and powdered by mortar and pestle to obtain a fine powder consisting of a CeO_2_ NPs-mediated okra extract.

### 2.3. Characterization

The characterization of CeO_2_ NPs provides a complete assessment of the morphology, crystalline size, stability, and most importantly functional groups liable to the bioreduction of the synthesized nanoparticles. The CeO_2_ NP, CS-CeO_2_ nanocomposite morphology was observed by SEM (JEOL, JSM-6480, Tokyo, Japan). The X-ray diffraction of CeO_2_ NPs was performed on PANalyticalX’Pert-PRO and interpreted using a matching software. A UV-visible spectrophotometer (Shimadzu, UV-2450, Tokyo, Japan) was utilized to acquire the absorption spectra of CeO_2_ NPs. CeO_2_ nanoparticles were also evaluated for the existence of biomolecules by Fourier transform infrared spectroscopy (FTIR, Thermo Fischer Scientific, Waltham, MA, USA). 

### 2.4. Antioxidant Activity 

Briefly, various concentrations of CeO_2_ NPs (20, 40, 60, 80 and 100 µg/mL) were added to 10 mL of a 0.1 mM 2,2-diphenyl-1-picrylhydrazyl (DPPH) solution and kept in complete darkness for at least 40 min at room temperature. Absorbance was recorded using ethanol as black at 517 nm wavelength. Ascorbic acid was used as the standard in all performing experiments. The antioxidant activity of green synthesized CeO_2_ NPs was evaluated by DPPH radical scavenging assay using the following formula [22]:

$\%\;Inhibition=\frac{Absorbance\;of\;Control-Absorbance\;of\;Sample}{Absorbance\;of\;Control}\times 100$

### 2.5. Cell Culturing and Exposure with CeO_2_ Nanoparticles

A T75 flask was used to culture HeLa cell lines. Hanks salt (10%) was supplemented with MEM (minimum essential medium), fetal bovine serum (FBS, 10 mL) and glutamine (2 mL). Moreover, the cells were incubated for 24 h at 37 ℃ to attain well interaction with the substratum. The cells were further sub-cultured once or twice every week. Then, the cells were composed using trypsin when the 75–85% convergence was obtained [23,24]. Furthermore, CeO_2_ NPs at the concentrations of 25–100 µg/mL was added for 24 h at 37 ℃ to the solution containing 5% carbon dioxide and 10% fetal bovine serum. 

### 2.6. In Vitro Measurement of Cellular Cytotoxicity (MTT Assay)

Human cervical carcinoma HeLa cells were added to a 96-well plate and then incubated in 5% CO_2_ at 37 ℃. Then, treated and untreated cells were exposed to increasing concentrations of CeO_2_ ranging from 10 to 125 μg/mL and allowed to incubate for 24 h. The MTT assay was controlled in each well; a total of 30 μL of MTT (20 mg/mL) was mixed and incubated for 4 h. Then, the medium was removed, and the cells were mixed in 100 μL of the DMSO solvent [24,25]. A microplate reader was used to measure the absorbance of the samples at 595 nm wavelength.

### 2.7. HeLa Cells Morphological Analysis

HeLa cells were put into a six-well plate. Each well contained 1 × 10^4^ cells and was treated with CeO_2_ NPs synthesized by the green extraction method with increasing concentrations for 12 h. The variation in the morphology of HeLa cells induced by CeO_2_ NPs were observed using 20× magnified inverted phase-contrast microscopy [23].

### 2.8. Antibacterial Study

The agar-well diffusion process was followed to analyze the bactericidal potential of green synthesized CeO_2_ NPs counter to *S. aureus* (Gram-positive) and *K. pneumonia* (Gram-negative) bacterial strains. First, the nutrient agar plates were prepared using 38 g of agar powder dissolved in 1 L of distilled water; then, the agar solution was autoclaved at 121 ℃ for 20 min. Then, it was cooled, mixed well, and poured into Petri plates (20 mL/plate); then, the agar plates were swabbed with bacterial strains [26]. At that time, these wells were exposed to 0.05 mL of the solution containing CeO_2_ nanoparticles at various concentrations. Then, these plates were incubated at 37 ℃ for 24 h. Then, the antibacterial activities of such nanoparticles were evaluated by measuring the inhibition zone around the wells.

### 2.9. Chitosan Hydrogel–CeO_2_ Composite Membranes Preparation

The chitosan-based hydrogel membranes were prepared by the freeze–gelation process. For this, a 2% solution of chitosan (CS) was prepared into the 1% acetic acid (25 mL). The green synthesized CeO_2_ nanoparticles (1% w.r.t chitosan) were suspended in deionized water via sonication for 5 min; then, the prepared suspension of CeO_2_ NPs was added to the CS solution, and the mixture solution was kept under continuous stirring for 2 h. Then, the glycerol (3 mL) was added up as a crosslinking material for CS, and the solution was kept in an oven at 80 ℃ for 4 h. After that, the homogeneous solution was cooled to normal temperature, poured into the Petri plate, and frozen at −20 ℃ for 2 days. Then, 3 M NaOH solution in ethanol was chilled at −20 ℃, poured onto the Petri plate of frozen chitosan, and frozen at −20 ℃ for 24 h. The prepared hydrogel was washed with ethanol (50%) three times and then distilled water until the neutralization (pH = 7) of the membrane. The same process was followed for the preparation of chitosan and 5% CeO_2_ NPs hydrogel membrane as dressings for wound treatment.

### 2.10. Wound-Healing Activity

#### 2.10.1. Swelling Percent of Chitosan Hydrogel–CeO_2_ Membranes

The membranes were cut into small and equal (1 cm × 1 cm) pieces for each sample in triplicate. The cut pieces were immersed in 4 mL of phosphate buffer saline (PBS) solution. The samples were taken out at a specific time interval (0.5 h, 2 h, 4 h, 8 h, 16 h, 24 h) and immediately weighed. The swelling percent of the membranes was evaluated by using the following formula:

$$Swelling\;\%=\frac{W2-W1}{W1}\times 100.$$

In expression, *W*1 represents the dry weight of the sample before immersion in solution and *W*2 represents the weight after immersion [27].

#### 2.10.2. Animal Trial

Male albino rats (9–10 weeks old and weighing approximately 220–250 g) were purchased. All animals were sustained at room temperature and given free access to water and food. All rat skin incision trials were conducted at Islamia University Bahawalpur with animal ethics committee number PAEC/21/45. All rats were divided into three groups, each group containing 4 rats randomly. Group one was treated with chitosan hydrogel membrane considered as the control group, group two was treated with chitosan hydrogel membrane loaded with 1% cerium nanoparticles, and group three was treated with 5% cerium oxide nanoparticles to evaluate the impact of green synthesized CeO_2_ NPs. All rats were anesthetized, and 2 cm full-thickness skin excision wounds were made. The prepared nanoparticles were locally applied at the wound place every day depending on the selected group. Finally, the wound diameter was measured every day [28].

### 2.11. Statistical Analysis

The outcomes were assessed as the mean ± standard deviation of 3 experiments. The data were assessed by Student’s *t*-test, and the values of *p* < 0.05 measured using the Excel software were statistically significant.

## 3. Results and Discussion

### 3.1. X-Ray Diffraction Analysis 

**Figure 1:**
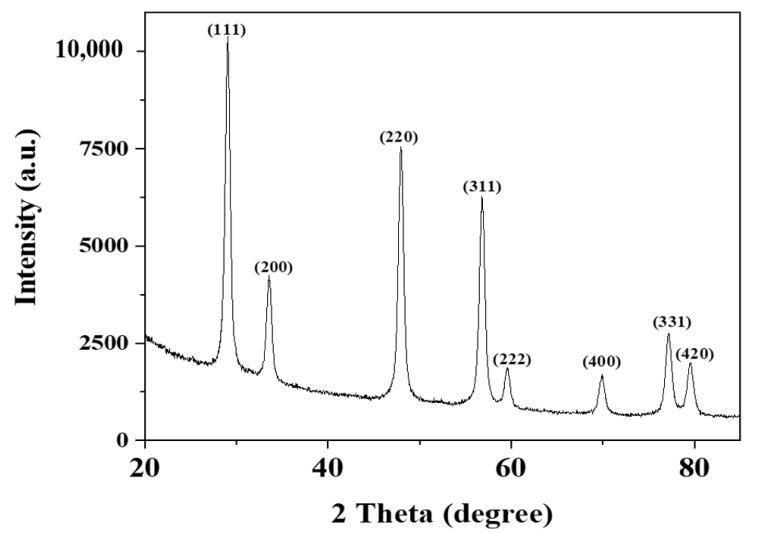
XRD patterns of CeO_2_ nanoparticles.

The XRD result of green synthesized CeO_2_ nanoparticles is shown in Figure 1. A strong and sharp diffraction peak revealed the crystalline nature of CeO_2_ nanoparticles. The indexed diffraction peaks at the 2θ position of 28.43°, 33.62°, 48.38°,57.74°, 59.03°, 69.37°, 76.69°, and 79.09°, corresponding to (111), (200), (220), (311), (222), (400), (331), and (420) crystal planes agreed with the COD library with the card no 96-900-9009 [29]. The XRD planes show the cubic structure for cerium dioxide nanoparticles with the lattice constant (a) value is 5.435 Å. The unit volume for green synthesized CeO_2_ mediated with *Abelmoschus esculentus* is calculated to be 160.54 Å^3^.

The crystallite size of the synthesized nanoparticles is calculated the Debye–Scherrer formula:

$$Average\;Crystallite\;Size\;\left(D\right)=\frac{0.9\lambda }{\beta \;cos\theta }.$$

In the formula, λ is the wavelength of the used X-ray for the analysis, β is the angular peak width at half maxima with units in radian, and θ represents the Bragg’s diffraction angle. The average crystallite size was approximately 30 nm using the above-mentioned mathematical relation.

### 3.2. SEM Analysis 

**Figure 2:**
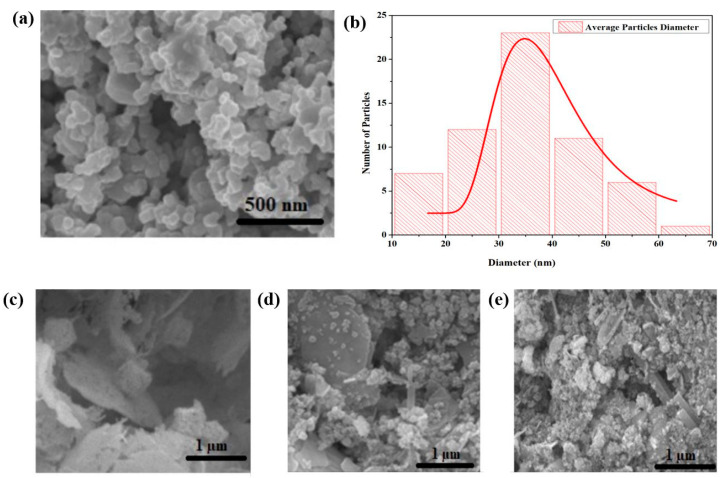
Surface morphology analysis by SEM: (**a**) green synthesized CeO_2_ NPs, (**b**) PSD analysis of CeO_2_ nanoparticles, (**c**) chitosan hydrogel membrane, (**d**) chitosan-loaded 1% CeO_2_ NPs; (**e**) chitosan-loaded 5% CeO_2_ NPs.

The SEM analysis of the prepared CeO_2_ nanoparticles using the *Abelmoschus esculentus* extract is shown in Figure 2a. The morphological observations revealed that green synthesized CeO_2_ NPs were fully homogenous. The average diameter of CeO_2_ NPs was calculated 36 nm by PSD analysis, as shown in Figure 2b using the Image J software. The particle size was approximately 35–40 nm, which almost confirmed the crystallite size determined by Scherer’s formula in XRD analysis [30,31]. 

Furthermore, SEM was utilized to investigate the surface morphology of the prepared chitosan (CS), CS-1% CeO_2_, and CS-5% CeO_2_ hydrogel membranes, as shown in Figure 2c,d. These SEM images exhibited the porous surface of the chitosan-based hydrogel membranes, these pores are interconnected to each other, and the nanoparticles are homogeneously distributed on the exterior of the hydrogel membranes.

### 3.3. UV-Visible Analysis 

**Figure 3:**
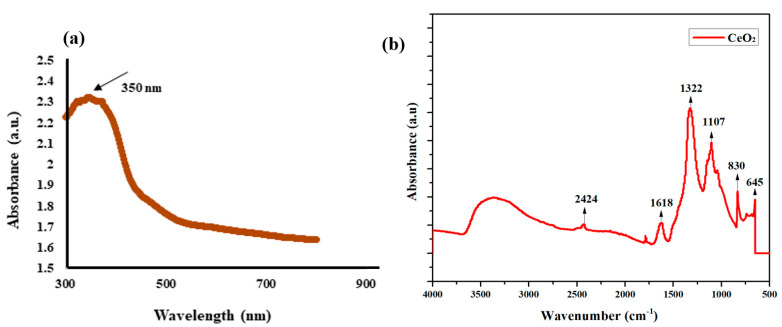
Green synthesized CeO_2_ nanoparticles (**a**) UV-visible and (**b**) FTIR spectroscopy.

The absorption spectrum of green synthesized CeO_2_ NPs measured by UV-vis spectroscopy is shown in Figure 3a. The maximum absorption band is located at 350 nm, which indicates the development of CeO_2_ nanoparticles. The presence of an expansion band also indicates the presence of oxidizing polyphenols, which are important for preventing the agglomeration of CeO_2_ nanoparticles [31]. 

### 3.4. FT-IR Analysis 

Figure 3b shows the FTIR results, which indicate that the stabilization of CeO_2_ NPs is due to the presence of phenolic and flavonoids compounds in the *Abelmoschus esculentus* extract. The peaks, which appeared at 3200–3500 cm^−1^, revealed the presence of the hydroxyl group (O-H) stretching frequency. The band at 645 cm^−1^ was ascribed to the Ce-O vibrations of green synthesized CeO_2_ NPs. The presence of minor distortions at 2424 cm^−1^ range indicates the presence of NH bonds in the *Abelmoschus esculentus* extract. The stretching vibration at 1322 cm^−1^ indicates the existence of the C–C bond, which implies the presence of polyphenol groups owing to the extract. In addition, the peak at 1618 cm^−1^ could be corresponded to the H-O-H group [32,33,34,35].

### 3.5. Antioxidant Activity of Green Synthesized CeO_2_ NPs Mediated Abelmoschus Esculentus

**Figure 4:**
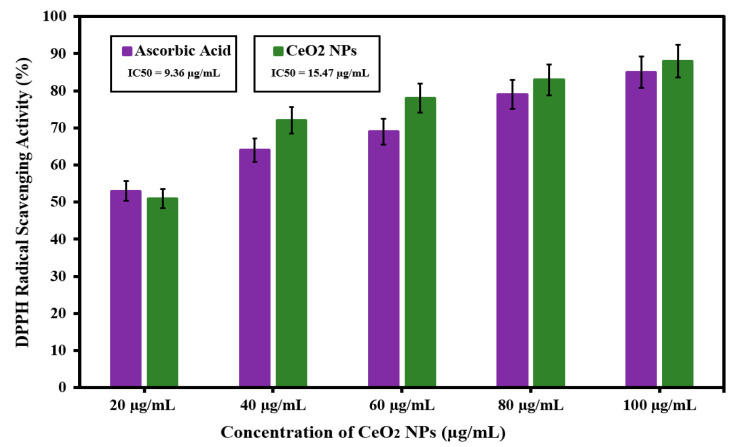
DPPH radical scavenging action of CeO_2_ nanoparticles and ascorbic acid.

In this study, the antioxidant activity of CeO_2_ NPs was highly dependent on the gradual increase in the concentration from 20 to 100 μg/mL of nanoparticles, as shown in Figure 4. For CeO_2_, the DPPH radical scavenging values were obtained (51.32%, 62.02%, 72.13%, 83.21%, and 88.15%) with the gradual increase in concentration of CeO_2_ nanoparticles. The investigations showed that CeO_2_ NPs mediated by the *Abelmoschus esculentus* extract showed better antioxidant properties than ascorbic acid. The ascorbic acid was used as the control to compare the antioxidant properties [36]. The antioxidant activity can be related to the transfer of electrons from oxygen atoms on the particle surface to the nitrogen atom of DPPH molecules, and DPPH forms stable molecules by accepting the electron. The IC50 values for ascorbic acid and CeO_2_ NPs were observed at 9.36 µg/mL and 15.47 µg/mL, respectively. Previous studies have shown that many metal nanoparticles can act as antioxidants and scavengers to free radicals [37]. It is also described that the antioxidant activity occurs mostly because of the high surface to volume ratio of the nanostructures. Correspondingly, zinc oxide NPs have greater scavenging action at higher concentrations [38]. However, recent results indicate that CeO_2_ NPs are excellent candidates as antioxidants.

### 3.6. Evaluation of Anticancer Activity of Green Synthesized CeO_2_ NPs Mediated Abelmoschus Esculentus 

**Figure 5:**
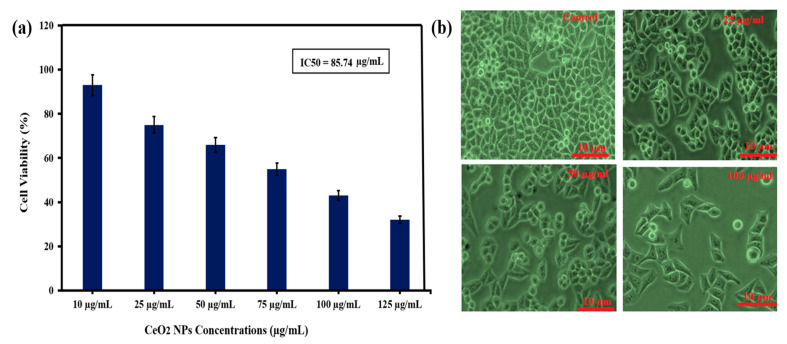
(**a**) Cell viability (%) of CeO_2_ treated HeLa cells after 24 h, (*t*-test). (**b**) Effect of green synthesized CeO_2_ NPs on HeLa cells at different doses (25, 75, and 100 µg/mL) and untreated cells (control).

HeLa cells were used to assess the cytotoxicity of CeO_2_ NPs using *abelmoschus esculentus* as a capping agent in the green synthesis method, and the MTT assay was used at different doses for 24 h. Figure 5a shows that the cell viability of 93%, 75%, 66%, 55%, 43%, and 33% decreased when HeLa cells were exposed to green synthesized CeO_2_ at increasing concentrations (10, 25, 50, 75, 100, and 125 µg/mL). The IC50 value was observed as 85.74 µg/mL for the Hela cells viability.

In addition, CeO_2_ NPs using the *Prosopis farcta* extract confirmed the cytotoxicity effects on the HT-29 cell line [39]. It was shown that the number of cells was reduced by employing the biosynthesized cerium oxide nanoparticles using carrageenan, which was highly dependent on the concentration of WEHI 164 malignant cells. In addition, the obtained data coincided with previous data, i.e., the loss in cell viability was dose/concentration-dependent [40,41,42,43,44].

In previous studies, it was suggested that the CeO_2_ NPs are toxic to cancerous cells but did not show any toxicity to the normal cells; also, higher concentrations have no toxic effect on the normal cells. A previous study also reported the use of 400 µg/mL CeO_2_ concentration for the evaluation of the anticancer effect against the human lungs cancer line. It can be suggested that the CeO_2_ NPs toxicity is specified only for cancerous cells and they may be safer for in vivo studies also in medicine and industry [45,46]. 

Furthermore, both cell morphology and cell viability assay results favored each other to encounter cervical HeLa cells. Figure 5b shows the photomicrograph of HeLa cells after approximately 24 h in the untreated (control) and presence of CeO_2_ NPs. In the control sample, the HeLa cells seem to have long-armed blurry cell borders that represent epithelial and bulky cells. In addition, with an increase in the concentration (25, 75, and 100 μg/mL) of green synthesized CeO_2_ nanoparticles during cervical HeLa cell treatment, a notable impact is observed compared to the control group. The nanoparticles-treated HeLa cells are considerably less dense and have contracted arms compared to the untreated (control) group, as shown in Figure 5b.

At lower concentrations (25 μg/mL), the cells started to shrink, and similarly, as the concentration increases (75 μg/mL), the further morphological changes could be seen, which indicate the apoptosis of cells. At 100 μg/mL, more apoptosis of the cancerous cells was observed. A similar pattern of anticancer was also observed for green synthesized CeO_2_ using orange peel extract [47]. 

### 3.7. Antibacterial Activity of Green Synthesized CeO_2_ NPs Mediated Abelmoschus Esculentus 

**Figure 6:**
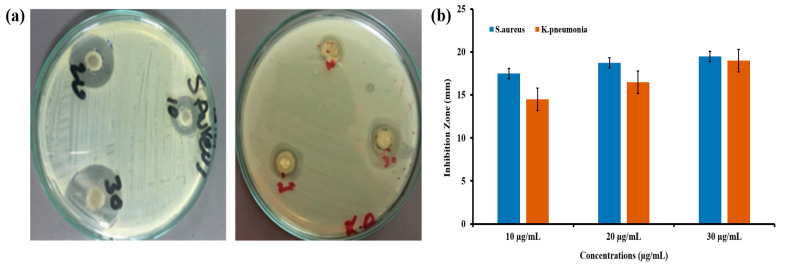
(**a**) Clear inhibition zone formed by synthesized CeO_2_ NPs using the *Abelmoschus Esculentus* extract against *S. aureus* and *K. pneumonia*. (**b**) Histogram representation for all measured inhibition zones at different concentrations of CeO_2_ NPs.

The antibacterial activity potential of green synthesized CeO_2_ NPs was tested against two pathogenic bacterial strains of *S. aureus* and *K. pneumonia* using the agar well diffusion method. The inhibition zones indicated the antibacterial effect of CeO_2_ nanoparticles at different concentrations. It was observed that the impact of increasing the concentration of CeO_2_ nanoparticles increased the inhibition of bacteria. Among three concentrations (10, 20, and 30 µg/mL), the 30 µg/mL of CeO_2_ nanoparticle concentration showed a higher inhibition zone against *S. aureus* (21 mm), which is Gram-positive, and *K. pneumonia* (19 mm), which is Gram-positive. Based on the results obtained by the agar well diffusion method, it is proposed that with an increase in the concentration of nanoparticles, the inhibition zone was increased at different concentrated doses for *S. aureus* and *K. pneumonia* strains, as shown in Figure 6a,b. This study shows that the antibacterial results of CeO_2_ NPs-medicated *abelmoschus esculentus* are vastly dependent on the concentration of nanoparticles; as the concentration was increased, the zone of inhibition also increased. From antibacterial study, it is seen that *S. aureus* (Gram-positive) bacteria are relatively more susceptible to CeO_2_ NPs than the *K. pneumonia* (Gram-negative) bacteria. It could be suggested that the Gram-positive bacteria are more sensitive to the reactive oxygen species (ROS), and this might be the reason of a higher zone of inhibition in the *S. aureus* bacterial strains. To compare our results, the same trend was also reported in green synthesized CeO_2_ mediated *Gloriosa Superba* leaves extract regarding the higher inhibition zone of Gram-positive bacterial strains [32]. 

The CeO_2_ potential against the microorganism greatly depends upon the greater surface to volume ratio, which causes the better interaction of nanoparticles with the surface of bacteria. In this study, we have synthesized nanoparticles with spherical morphology and excellent average diameter which, as discussed in SEM analysis, is highly correlated with the antibacterial potential. Moreover, the positive surface of the nanoparticle ions and negatively charged surface of the bacteria causes a strong electrostatic interaction, which ruptures the outer surface of the bacteria and causes cell death.

The basic mechanism of the antibacterial activity of the CeO_2_ NPs is that when the light falls on the nanoparticle surfaces, they absorb the photo-energy greater than the CeO_2_ bandgap. Then, the electrons (e^−^) move from the valence band to the conduction band, and holes (h^+^) are generated in the valence band. The photo-generated electrons and holes cause reduction and oxidation processes. The electron reacts with oxygen and produces the superoxide anion (O_2_**^●^**^−^), and this is known as the reduction process. Furthermore, the holes react with the hydroxyl ions and produce the hydroxyl radical (**^●^**OH) by the oxidation process. Hydroxyl radicals are a very reactive oxidant to the organic biomolecules such as DNA nucleic acid, proteins, and lipids. The interaction of superoxide anions in water contents produces indirectly singlet oxygen (^1^O_2_), which is the mean mediator of the phototoxicity caused by the damage of treated tissues. These photo-generated hydroxyl radicals such as superoxide anion and singlet oxygen known as the reactive oxygen species (ROS) which reach through their direct interaction with the cell wall and penetration to the internal part of the bacteria destroy the DNA, proteins, and lipids functions, leading to the death of bacterial cells. In addition, the mechanical damage by the direct interaction of the CeO_2_ NPs with the bacterial surface causes the denaturing [48].

### 3.8. Wound-Healing Potential of Green Synthesized CeO_2_ NPs Mediated Abelmoschus Esculentus

#### 3.8.1. Swelling Studies of Hydrogel Membrane 

**Figure 7:**
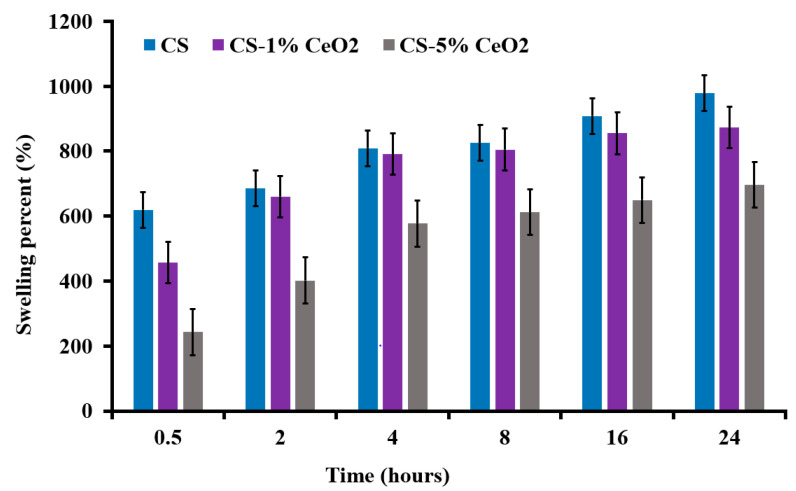
Demonstration of the swelling percent of the chitosan-based hydrogel membranes.

Fluid such as plasma or serum from the wound absorbed by the dressing has a key role in wound healing. So, it is important to know about the swelling capacity of the prepared hydrogel-based dressings for the wound. Figure 7 represents the time-dependent swelling behavior of the chitosan (control) and chitosan-loaded 1% and 5% green synthesized CeO_2_ nanoparticles. The chitosan hydrogel membrane represents the highest swelling capacity. In the other chitosan hydrogel membrane incorporated with CeO_2_ NPs, it was evaluated that the swelling capacity of the membrane dressing decreases with the cumulative concentration of the nanoparticles. There was a decrease in the swelling capacity of the membranes due to the higher crosslinking; the higher crosslinking occupies the hydrophilic functional sites available in the hydrogel matrix for intermolecular bonding [49].

#### 3.8.2. Animal Trials

**Figure 8:**
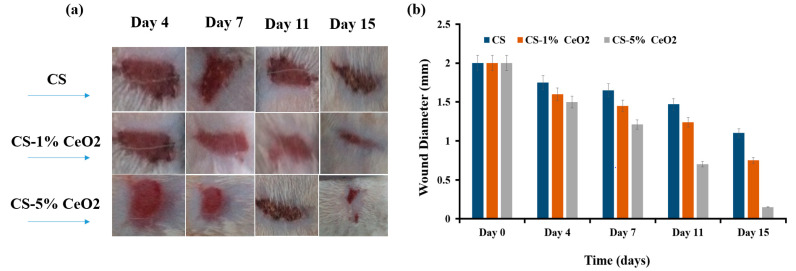
(**a**) Photographs representing the analysis of incision wound treatment with green synthesized CS-1% CeO_2_ and CS-5% CeO_2_ nanoparticles incorporated in chitosan hydrogel membrane groups. (**b**) Comparison of wound reduction in diameter.

Figure 8a shows the fine changes in wound extent during the wound-healing development for different samples of green synthesized CeO_2_. The healing procedure was assessed by observing regular changes in the color of the wound. The photographs of the representative rat wounds from each group were acquired to evaluate the healing potential of the cerium oxide nanoparticles at different concentrations. After 4 days of healing, fresh skin was grown (after it was treated with 1% and 5% cerium oxide nanoparticles); it had fewer scabs and was smoother than the control group; these observations represent the initiation of the wound-healing process. On day 7, a brown dark color was observed for the cerium oxide nanoparticles-treated groups. These observations confirm the initiation of wound-healing progress by producing collagen. On day 11, the 1% and 5% cerium oxide nanoparticles-treated group showed that the wounds were significantly reduced in size compared to the control group. Regarding the scab formation of the control group, an increase in the concentration of cerium oxide nanoparticles significantly affected the wound size. 

The wound closure comparison of incision is shown in Figure 8b, which represents the effect of the concentration of cerium oxide nanoparticles on the diameter of the wound compared with the control sample. On day 15, the wound site differed from the treated group in which the maximum contraction of the wounds was achieved by increasing the concentration of the cerium nanoparticles as compared to the control group, which showed a visibly larger wound size.

According to these observations, it can be concluded that CS-1% CeO_2_ and CS-5% CeO_2_ nanoparticles incorporated in chitosan hydrogel membrane groups stimulate the wound-healing process specifically during the initial stages. In the wound-healing experiment in vivo, the groups treated with cerium oxide nanoparticles incorporated in chitosan hydrogel membrane showed better wound healing after 11 days of excision. As discussed above, green synthesized cerium oxide nanoparticles have exceptional antibacterial properties, which considerably affect the wound-healing process. Recent studies have also reported that metal oxide nanoparticles reduce bacterial contamination, increase collagen formation and tensile strength, and decrease wound inflammation. In addition, the increasing concentration of nanoparticles affected the regulation of fibrogenic cytokines, which greatly contributed to the healing process [50]. 

## 4. Conclusions

Cerium oxide nanoparticles were green synthesized and characterized by manifold techniques (UV-visible spectroscopy, SEM, XRD, and FTIR). Furthermore, CeO_2_ NPs have resilient antioxidant activity, while MTT assay outcomes and morphological analysis after 24 h indicated that CeO_2_ NPs demonstrated a clear cytotoxic effect against HeLa cell lines. CeO_2_ NPs were considered to be associated with a cell-killing mechanism when used at higher doses (50–125 µg/mL). The green synthesized cerium oxide nanoparticles showed higher antioxidant activities and bactericidal effect against both Gram-positive and Gram-negative strains. The in vivo studies on rats confirmed that green synthesized cerium oxide nanoparticles appeared to be effective for skin wound treatment. The wound treatment with cerium nanoparticles induced collagen deposition and increased the tensile strength of skin as compared to the control group. This study may serve as a fruitful platform to explore the green synthesis of nanoparticles in various biomedical therapeutics.

## Data Availability

All relevant data are included in the article.

## Sample Availability

Samples of the compounds are available from the authors.
